# The utility of recombinant factor VIIa as a last resort in trauma

**DOI:** 10.1186/1749-7922-7-S1-S7

**Published:** 2012-08-22

**Authors:** Rishi Mamtani, Bartolomeu Nascimento, Sandro Rizoli, Ruxandra Pinto, Yulia Lin, Homer Tien

**Affiliations:** 1 Sunnybrook Health Sciences Centre, 2075 Bayview Avenue, Room H113, Toronto, ON M4N 3M5, Canada; 2 Trauma Program, Department of Surgery, Sunnybrook Health Sciences Centre, 2075 Bayview Avenue, Room B5 12, Toronto, ON M4N 3M5, Canada; 3 Departments of Surgery and Critical Care Medicine, Sunnybrook Health Sciences Centre, University of Toronto, Canada; 4 Sunnybrook Health Sciences Centre, 2075 Bayview Avenue, Room K3W-25, Toronto, ON M4N 3M5, Canada; 5 Sunnybrook Health Sciences Centre, 2075 Bayview Avenue, Room B2 04, Toronto, ON M4N 3M5, Canada; 6 Trauma Services, Division of General Surgery, Sunnybrook Health Sciences Centre and Canadian Forces Health Services, 2075 Bayview Avenue, Room H1 86, Toronto, ON M4N 3M5, USA

## Abstract

**Introduction:**

The use of recombinant factor VII (rFVIIa) as a last resort for the management of coagulopathy when there is severe metabolic acidosis during large bleedings in trauma might be deemed inappropriate. The objective of this study was to identify critical degrees of acidosis and associated factors at which rFVIIa might be considered of no utility.

**Methods:**

All massively transfused (≥ 8 units of red blood cells within 12 hours) trauma patients from Jan 2000 to Nov 2006. Demographic, baseline physiologic and rFVIIa dosage data were collected. Rate of red blood cell transfusion in the first 6 hours of hospitalization (RBC/hr) was calculated and used as a surrogate for bleeding. Last resort use of rFVIIa was defined by a pH≤ 7.02 based on ROC analysis for survival. In-hospital mortality was analyzed in last resort and non-last resort groups. Univariate analysis was performed to assess for differences between groups and identify factors associates with no utility of rFVIIa.

**Results:**

71 patients who received rFVIIa were analyzed. The pH> 7.02 had 100% sensitivity for the identification of potential survivors. All 11 coagulopathic, severely acidotic (pH ≤ 7.02) patients with high rates of bleeding (4RBC/hr) died despite administration of rFVIIa. The financial cost of administering rFVIIa as a last resort to these 11 severely acidotic and coagulophatic cases was $75,162 (CA).

**Conclusions:**

Our study found no utility of rFVIIa in treating severely acidotic, coagulopathic trauma patients with high rates of bleeding; and thus restrictions should be set on its usage in these circumstances.

## Introduction

Recombinant Factor VIIa (rFVIIa; Novoseven®, NiaStase®) is a hemostatic agent licensed for the management of hemorrhagic events and averting bleeding during invasive interventions in hemophilia A and B patients with FVIII and FIX inhibitors; acquired hemophilia; congenital deficiency of factor VII; and for the treatment of Glanzmann’s thrombasthenia [1-3]. It has also been used off-label and studied in the treatment of coagulopathy in trauma patients [4-7].

The use of rFVIIa for non-approved indications has been formally evaluated in clinical trials (including two randomized controlled trials in trauma) [8-10], and shown to be of no survival benefit [11]; and with clear evidence of harm, particularly in the elderly [12]. Despite the lack of supporting evidence, transfusion guidelines in either military or civilian settings currently suggest the use of rFVIIa as a last resort for the management of refractory coagulopathy in trauma [13-16]. However, when the drug is used in these settings of massive hemorrhage, its efficacy as a pro-hemostatic agent may vary under different physiologic conditions, particularly in acidosis [17,18]. In metabolic acidosis, when pH levels are under 7.2, the activity of rFVIIa is significantly stunted. In fact, an investigation conducted by Meng et al. indicated that the activity of rFVIIa decreased by over 90% at a pH level of 7.0 [17]. Furthermore, high expenditures are associated with off-label use of rFVIIa [19]. Therefore, the use of rFVIIa as a last resort when there is severe metabolic acidosis during significant hemorrhage in trauma might be considered inappropriate.

We reviewed a cohort of massively transfused trauma patients to whom rFVIIa was administered to evaluate its utility as a last resort for the management of traumatic coagulopathy. The objective of this study was to identify critical degrees of acidosis and associated factors at which the use of rFVIIa might be considered of no utility.

## Methods

This study was conducted at Tory Regional Trauma Centre of Sunnybrook Health Sciences Centre (SHSC), a large Canadian Level I adult trauma facility. The study protocol was reviewed and approved by the Hospital Research Ethics Board.

### Study cohort

Patient information was obtained from the Blood Bank information system (HCLL, Mediware, N.Y.) at SHSC and the computerized Trauma Registry. The cohort was comprised of patients admitted from January 1, 2000 to November 30, 2006, with the following inclusion criteria: (1) having been massively transfused, defined as having received 8 or more units of red blood cells (RBCs) within the first 12 hours (h) of admission (analogous to established criterion in recent randomized control trials on rFVIIa in trauma) [8,9]; (2) having received rFVIIa; (3) having recorded pH values; (4) and having recorded times during which dosages of rFVIIa were administered (from admission to administration).

Last resort use of rFVIIa was defined based on Receiver Operating Characteristics (ROC) curve analysis for survival. The ROC curve was determined to define a specific pH cutoff at which the test could appropriately discriminate the two groups based on the highest sensitivity for identifying potential survivors. The group with low survival based on the pH cutoff was defined as the group receiving last resort use of rFVIIa.

### Data collection

Demographic data were obtained from the Trauma Registry and included the following: age, gender, type of injury, Abbreviated Injury Scale (AIS) score, Injury Severity Score (ISS), and note of discharge or in-hospital mortality. Electronic patient records and manual chart abstraction were used to gather data on in-hospital mortality and admission laboratory values including: platelet counts, hemoglobin level, arterial pH, International Normalized Ratio (INR), and plasma fibrinogen levels. The Blood Bank Information System (HCLL, Mediware, N.Y.) was used to determine patients who received rFVIIa for coagulopathy treatment within the first 24h of admission. The same database was utilized to obtain the time that RBC units were provided, and this information was verified by the hospital chart. The rate of transfusion for the first 6h of hospitalization was determined for all patients in the cohort. In our previous experience, this variable, used as a surrogate marker of the severity of bleeding, has shown to strongly predict 24h in-hospital death [20,21]. The rate of transfusion is also indicative of severity of injury and the urgency of treatment.

The price quote of the supplies of rFVIIa was obtained from the manufacturer and a recently published cost-effectiveness analysis [19,22]. We conducted cost analysis pertaining to the drug’s administration as a last resort. We reviewed the monetary prices of rFVIIa dosages in the acidotic patients who died despite receiving the drug.

### Outcome measures

The main outcome measure was in-hospital mortality. Secondary outcomes were patient’s physiological covariates (ISS, AIS for head injury, gender, age, fibrinogen, rate of RBC transfusion within 6h of hospitalization and INR). The impact of rFVIIa administration was assessed by comparing outcomes between last resort and non-last resort cases. Also, sensitivity, specificity, negative predictive value (NPV) and positive predictive value (PPV) were calculated in relation to pH (defined by the best sensitivity on ROC cut-off for survival) and in-hospital mortality. An additional outcome measure was direct monetary costs associated with the use of rFVIIa for cases deemed inappropriate.

### Statistical analysis

The main variables present in this study were pH and in-hospital mortality. Other covariates included pertained to the patient’s physiological state (ISS, AIS for head injury, gender, age, base deficit, lactate, fibrinogen, rate of RBC transfusion within 6h of hospitalization and INR).

Last resort use of rFVIIa was defined based on ROC analysis for survival as aforementioned. The ROC curve was determined to define a specific pH cutoff at which the test could appropriately discriminate the two groups based on survival. From this value, the sensitivity, specificity, PPV and NPV were derived.

Potential determinants of rFVIIa failure were analyzed through a subgroup analysis of baseline characteristics, including degree of shock and acidosis, age, ISS, coagulopathy, rFVIIa dose regimens, and rates of RBC transfusion.

Continuous variables were expressed in standard deviations, medians, means, or interquartile ranges (IQR); these were compared using T-test or Mann-Whitney U test. Categorical variables were presented as percentages, and compared using chi-square or Fisher’s exact test. All analyses were performed using SAS 9.1 (SAS Institute Inc., Cary, NC). Two-sided p values were used and statistical significance was set at p < 0.05.

## Results

A total of 7,076 patients were seen by the Sunnybrook trauma team during the 6-year study period. Within this group, 328 (4.6%) patients were massively transfused. Of these, 72 (22%) patients received rFVIIa. One patient was excluded due to absent pH data. Upon further investigation, it was noted that this subject had a low numerical ISS score, blunt trauma with no head injury, and received only one dose of 200 µg/kg of rFVIIa, given after 6.9 h in the hospital. He remained stable throughout his hospital stay. Therefore, our study cohort consisted of 71 massively transfused patients who received rFVIIa and had known pH values, meeting our entry criteria. All 71 patients had complete data sets for all variables studied.

The area under the ROC curve analysis for pH and survival was approximately 0.70 for the pH value 7.02, which had the highest sensitivity to identify survivors. The sensitivity of pH > 7.02 to identify survival was 100% and specificity of pH ≤ 7.02 for in-hospital mortality was 100%. The PPV was 56.7% and the NPV was 100%. The use of this best cut-off for pH based on the ROC curve for our subgroup analysis is supported by previous research suggesting that the efficacy of rFVIIa decreases by 90% when the body pH decreases from 7.4 to 7.0 [17]. Therefore, we divided our cohort into 2 groups based on admission pH (patients with pH ≤ 7.02 were analyzed in the last resort group while patients with pH > 7.02 in the non-last resort group). Clinical characteristics and demographics of the entire study cohort and subgroups based on pH are summarized in Table 1. Overall, there were no significant differences between the two subgroups with respect to age, gender, type of injury, ISS, Head AIS, and dose of rFVIIa given. Baseline coagulation profiles showed significant differences in platelets (p < 0.01) and INR (p = 0.03), except for fibrinogen (p = 0.07). Additionally, the rate of bleeding using transfusion as a surrogate marker was significantly higher in the severely acidotic group (4 RBC units per hour ± 1.5 vs. 3 ± 1.7; p=0.03).

**Table 1:** Demographics & Baseline Characteristics

Variable	Last resort (n=11)	Non-last resort (n=60)	P Value
Age (years)	27 (22, 39)	35 (24, 48)	0.14
Male (%)	82	63	0.3
Penetrating (%)	45	28	0.2
ISS	47 (±16)	43(±15)	0.4
Head AIS	0 (0, 2)	2 (0, 5)	0.1
Platelets	76 (±57)	184 (±95)	<0.01
Fibrinogen	0.64 (±0.3)	0.9 (±0.5)	0.07
INR	2.1 (1.8,2.7)	1.4(1.2, 1.6)	0.03
Hemoglobin	83 (±17)	100 (±28)	0.02
pH	6.87 (±0.11)	7.26 (±0.11)	<0.01
Rate of Bleeding (RBC/hr)	4 (±1.5)	3 (±1.7)	0.03
Time to rFVIIa (hr)	3.7 (±2.2)	6.2 (4.5)	0.04
rFVIIa Dose (ug/Kg)	89 (±43)	116 (±79)	0.14
> 1 rFVIIa doses (%)	9	33	0.05

Values are presented as mean (±SD) or median (IQR - Interquartile Range) when appropriate. ISS, injury severity score; AIS, abbreviated injury scale; INR, international normalized ratio; RBC/hr, units of red blood cells per hour in the first 6 hrs of admission; Statistical significance was set at p<0.05

A comparison of mortality between the two groups is shown in Table 2. Of the 11 severely acidotic (pH ≤ 7.02) patients in the last resort group, all (100%) died. Of the 60 less acidotic (pH > 7.02) patients in the non-last resort group, 26 (43%) died.

**Table 2:** pH & In-hospital Mortality

	Alive	Dead	Hospital Mortality
pH > 7.02 (n=60)	34	26	43%
pH ≤ 7.02 (n=11)	0	11	100%
**Sensitivity** 100% (34/34)	**Specificity** 30% (11/37)	**(PPV)** 57% (34/60)	**(NPV)** 100% (11/11)

PPV, positive predictive value; NPV, negative predictive value

The vast majority, 72% of rFVIIa-treated patients received only 1 dose, while 24% received 2 doses, and 4% received 3 doses after being admitted to the hospital. The first dose was administered after a median time interval of 4.5h (2.7, 7.7). Repeated doses were administered after an average time interval of 2.3h. This indicated that as the patient’s condition deteriorated, more doses of rFVIIa were administered in an expedited fashion. The median initial dose was 85.7µg/kg (61.6, 102.8). This was also the overall median dosage, as most patients only received 1 dose.

Of note, a transfusion medicine specialist at SHSC approved the use of rFVIIa as a final alternative when all potential interventions failed. In the years 2000 and 2001, low doses of 17.1µg/kg of rFVIIa were administered after patients received more than 20 units of RBCs. However, following a supportive randomized control trial on rFVIIa in trauma [8], fewer units of RBCs were noted to be transfused prior to rFVIIa administration and more doses of rFVIIa were given from 2002 onwards.

The total cost of administrating sufficient doses of rFVIIa to the 11 patients as a last resort was approximately $75,162 (CA). This monetary cost was measured solely based on the amounts of doses of rFVIIa given and excluded other expenditures associated with the administration of the drug. In the United States of America, a low dose (1,200 µg or 17.1µg/kg on a 70 kg average adult) of rFVIIa is the smallest available unit dose that costs approximately the same as 8 units of plasma [23]. The price of one unit of plasma is approximately $120 (USD), including expenditures related to administering them [23].

## Discussion

Over the last decade, rFVIIa has been explored as a potential treatment for many coagulopathic states other than congenital conditions and hemophilias [7,11,24] . Theoretically, rFVIIa seems to be an appealing option following trauma because of its systemic intravenous administration and swift mechanism of action, acting at the injury site by binding to exposed tissue factor, and expediting the generation of thrombin on activated platelets to propel the coagulation cascade forward. However, in the context of massive hemorrhage, there are potential limiting factors such as acidosis and refractory shock.

From this study, a pH of 7.02 had the best sensitivity on the ROC curve for discriminating survivors and non-survivors. A pH > 7.02 was 100% sensitive at identifying potential survivors, reassuring the clinician that no probable survivors could have been missed if this pH cut-off was adopted. Thus, a pH of 7.02 may be used as a potential guideline or measure at which the administration of rFVIIa should not be considered for patients who are severely acidotic. The pH level of these patients appeared to be a key determining factor in the success of rFVIIa. As noted, there was a remarkable 100% mortality noted in coagulopathic and severely acidotic patients (pH ≤ 7.02) who had high bleeding rates, despite the use of rFVIIa. This is corroborated by recent research suggesting that the efficacy of rFVIIa decreases by 90% when the body pH decreases from 7.4 to 7.0 [17]. However, in a recent animal model of lactic acidosis, the effectiveness of rFVIIa in correcting abnormal INR values at a mean pH of 7.14 was unaffected [18]. This suggests that other factors may influence its efficacy in clinical settings.

In keeping with our findings, data from the Australia and New Zealand Haemostasis Registry on 10 years of the use of rFVIIa in Australia and New Zealand which reports on the outcomes of 2181 trauma cases, the single most important predictor of the effect of rFVIIa on bleeding and 28-day mortality was pH [25]. In their multivariate analysis, for every 0.1 decline in pH, there were associated increases in non-responders to rFVIIa use and mortality rates [25]. Their unadjusted analysis on the relationship between 28-day mortality and pH showed that patients with pH < 6.90 had a mortality rate of 98% while the group with 7.30<pH<7.39 had a mortality of 23% [25]. Although the pH of 6.90 did not coincide with our threshold of 7.02, the pattern is apparent that mortality percentage drastically increases with decreases in pH. Logistic regression analysis was conducted and values for the odds ratio were obtained for the effect on bleeding and pH, as well as 28-day mortality and pH. For both, an inverse correlation was seen, in that when pH decreased, the odds ratio for mortality increased [25]. Furthermore, outside of the trauma literature, a study by Karkouti et al. found that the administration of rFVIIa should be expedited in order to increase its efficacy in cardiac surgery [24].

An additional factor that must be considered is the impact of other variables, such as rate of bleeding and baseline physiologic factors on rFVIIa, particularly temperature. Hypothermia is a well-known complication seen in the natural progression of traumatic injury, blood loss, hypovolemia, and shock [26]. While our study identifies correlations of pH with the effectiveness of rFVIIa, a recently conducted study by Meng et al., suggests that a decrease in temperature from 37°C to 33°C also results in a reduction of rFVIIa’s activity by 20% [17]. The Australia and New Zealand Haemostasis Registry also presented graphical data pertaining to the effect of decreases in temperature and response of bleeding to rFVIIa administration in trauma patients. In fact, for ≤ 33.5°C, 70.7% of trauma patients had an unchanged bleeding response; and for normal physiologic temperature range (36.6-37.5°C), 38% had an unchanged bleeding response after receiving rFVIIa [25]. The registry also found that as pH is decreased, the activity of rFVIIa is reduced [25]. Finally, a study by Knudson et al analyzed subgroup of patients who received rFVIIa and lived at least 24 hr versus those who received rFVIIa and died. In this study, predictors of death included a low pH, a low platelet count, a more severe base deficit, and a higher transfusion rate [27]. In our present study, higher transfusion rates were also associated with failure of rFVIIa and increased mortality. These findings indicate that the efficacy of rFVIIa in coagulopathic, acidotic patients with high rates of bleeding is compromised with pH and temperature reductions.

As the patient’s condition deteriorates over time due to failure of standard therapies, the pH drastically decreases and the activity of rFVIIa is virtually nonexistent, which makes it a challenge to consider the use of rFVIIa as a last resort. Thus, current recommendations on its use as an alternative to manage coagulopathy in trauma when other interventions fail should be taken with caution.

The high monetary cost of rFVIIa administration, with no strong evidence of survival benefit [7,11] and increased risks of thrombotic complications [12], also calls for a review of guidelines recommending the use of this medication for traumatic coagulopathy. The cost-effectiveness of using rFVIIa as a last resort therapy for critical bleeding requiring massive transfusion was recently evaluated [19]. The incremental costs of rFVIIa increased with severity of illness and transfusion requirement, and were unacceptably high (> US$100,000 per life-year) for most patients [19]. Overall, thought must be given to the expense of rFVIIa, and its utility as a last resort.

Alternatively, a more affordable and effective management strategy for traumatic coagulopathy is available. A recently conducted large randomized control trial (CRASH-2) involving 20,000 patients found that tranexamic acid reduced the risk of death in hemorrhaging trauma patients and should be recommended in bleeding trauma situations [28]. International cost-analyses supporting the use of tranexamic acid as opposed to administering rFVIIa found that the cost of giving tranexamic acid compared to not giving it was $18,025 in Tanzania, $20,760 in India and $48,002 in the UK [29]. The case being made for increased administration of tranexamic acid is bolstered by the lack of increased thromboembolic events observed in the CRASH-2 trial. In Total Knee Arthroplasty (TKA), a reduction in the number of blood transfusions has also been observed with no increase in symptomatic thromboembolic phenomena [30]. Tranexamic acid may not only be helpful from a biological perspective, but also in a monetary manner, in reducing resources in obtaining and providing blood products [30,31].

### Limitations

The main limitations of this study are its retrospective nature, small size of the severely acidotic (pH ≤ 7.02) subgroup, and the changes over time with respect to the use of rFVIIa. Towards the start of the study period, this drug was dosed as low as 17.1µg/kg, and was considered as a final alternative therapy. However, further to research advances at the time, a shift towards increased doses and earlier use was noted by the year 2002, which continued to evolve until the end of the study period. This may also have had some impact upon observed results. The pH data reflects the patient’s condition on arrival, which might not represent changes in degrees of acidosis immediately before the administration of the drug. However, the drug was administered only 3.7h after admission for the severely acidotic group and 6.2h for the less acidotic patients when other standard therapies had failed; thus a worsening pH level is intuitively expected in these clinical situations. The area under the ROC curve was tabulated to be 0.70, indicating potential for a more accurate cutoff for determining at which pH range the administration of rFVIIa should be more reserved. Finally, we did not have information on all co-morbidities that may have contributed to mortality.

## Conclusions

Our study found no utility of rFVIIa in treating coagulopathic trauma patients with pH ≤ 7.02 and high rates of bleeding (4 units of RBC/h); and thus restrictions should be set on its usage in these circumstances. Furthermore, the lack of evidence demonstrating any survival benefit of rFVIIa in trauma, in conjunction with the potential increased risk of thromboembolic complications and high monetary costs of its off-label use, renders its utility highly questionable in such situations.

Future research should be conducted in finding alternatives to rFVIIa in the management of trauma coagulopathy. We hope our findings will guide physicians when deciding on the inclusion of this drug as part of massive transfusion protocols in trauma.

## Abbreviations used

RBC: Red Blood Cell; rFVIIa: Recombinant Factor 7a; AIS: Abbreviated Injury Score; ISS: Injury Severity Score; INR: International Normalized Ratio.

## Competing interests and disclaimer

BN is the recipient of the 2010 National Blood Foundation Grant for the conduct of research related to coagulopathy in trauma. SR has been a consultant for Novo-nordisk, the manufacturer of Recombinant FVIIa. YL is a site investigator for a registry on the off-label use of recombinant factor VIIa that is funded by an unrestricted educational grant from Novo Nordisk. The other authors have no conflict of interest to declare.

## Authors' contributions

RM participated in the writing of the manuscript and was responsible for following the final submission guidelines. BN contributed to the study design; data collection and analysis; writing of the manuscript; and manuscript review. SR participated in the study design; its writing; and review. RP provided statistical support and reviewed the manuscript. YL participated in the writing and review of the manuscript. HT participated in the study conception; its writing; and review.

## References

[B1] Hedner U Mechanism of action, development and clinical experience of recombinant FVIIa J Biotechnol 2006 124 4 747 57 Epub 2006 May 12. Review 10.1016/j.jbiotec.2006.03.042 16697480

[B2] Parameswaran R Shapiro AD Gill JC Dose effect and efficacy of rFVIIa in the treatment of haemophilia patients with inhibitors: analysis from the Hemophilia and Thrombosis Research Society Registry Haemophilia 2005 11 2 100 6 10.1111/j.1365-2516.2005.01075.x 15810910

[B3] Hedner U Recombinat factor VIIa: its background, development and clinical use Curr Opin Hematol 2007 14 225 9 doi: 10.1097/MOH. 0b013e3280dce57b 10.1097/MOH.0b013e3280dce57b 17414211

[B4] Kenet G Walden R Eldad A Treatment of traumatic bleeding with recombinant factor VIIa Lancet 1999 354 9193 1879 10.1016/S0140-6736(99)05155-7 10584732

[B5] Martinowitz U Kenet G Lubetski A Possible role of recombinant activated factor VII (rFVIIa) in the control of hemorrhage associated with massive trauma Can J Anaesth 2002 49 10 S15 20 12546003

[B6] Mohr AM Holcomb JB Dutton RP Recombinant activated factor VIIa and hemostasis in critical care: a focus on trauma Crit Care 2005 9 Suppl 5 S37 42 Epub 2005 Oct 7 10.1186/cc3784 16221318 PMC3226122

[B7] Barletta JF Ahrens CL Tyburski JG A review of recombinant factor VII for refractory bleeding in nonhemophilic trauma patients J Trauma 2005 58 3 646 51 10.1097/01.TA.0000154561.97961.AD 15761369

[B8] Boffard KD Riou B Warren B NovoSeven Trauma Study Group. Recombinant factor VIIa as adjunctive therapy for bleeding control in severely injured trauma patients: two parallel randomized, placebo-controlled, double-blind clinical trials J Trauma 2005 59 1 8 15 discussion 15-8 10.1097/01.TA.0000171453.37949.B7 16096533

[B9] Hauser CJ Boffard K Dutton R CONTROL Study Group. Results of the CONTROL trial: efficacy and safety of recombinant activated Factor VII in the management of refractory traumatic hemorrhage J Trauma 2010 69 3 489 500 10.1097/TA.0b013e3181edf36e 20838118

[B10] Dutton RP Parr M Tortella BJ Recombinant Activated Factor VII Safety in Trauma Patients: Results from the CONTROL Trial J Trauma 2011 71 1 12 19 10.1097/TA.0b013e31821a42cf 21610529

[B11] Lin Y Stanworth SJ Birchall J Recombinant factor VIIa for the prevention and treatment of bleeding in patients without haemophilia Cochrane Database Syst Rev 2011 2 CD005011 21328270 10.1002/14651858.CD005011.pub3

[B12] Levi M Levy JH Andersen HF Safety of recombinant activated factor VII in randomized clinical trials N Engl J Med 2010 363 19 1791 800 Erratum in: N Engl J Med. 2011 Nov 17;365(20):1944 10.1056/NEJMoa1006221 21047223

[B13] Wade CE Eastridge BJ Jones JA Use of recombinant factor VIIa in US military casualties for a five-year period J Trauma 2010 69 2 353 9 10.1097/TA.0b013e3181e49059 20699744

[B14] Woodruff SI Dougherty AL Dye JL Use of recombinant factor VIIA for control of combat-related haemorrhage Emerg Med J 2010 27 2 121 4 10.1136/emj.2008.060657 20156864

[B15] Rossaint R Bouillon B Cerny V Management of bleeding following major trauma: an updated European guideline Crit Care 2010 14 2 R52 10.1186/cc8943 20370902 PMC2887168

[B16] Vincent JL Rossaint R Riou B Recommendations on the use of recombinant activated factor VII as an adjunctive treatment for massive bleeding--a European perspective Crit Care 2006 10 4 R120 10.1186/cc5026 16919168 PMC1750973

[B17] Meng ZH Wolberg AS Monroe DM 3rd The effect of temperature and pH on the activity of factor VIIa: implications for the efficacy of high-dose factor VIIa in hypothermic and acidotic patients J Trauma 2003 55 5 886 91 10.1097/01.TA.0000066184.20808.A5 14608161

[B18] Lesperance RN Lehmann RK Harold DM Recombinant Factor VII is Effective at Reversing Coagulopathy in a Lactic Acidosis Model J Trauma 2011 [Epub ahead of print] 10.1097/TA.0b013e318224e24a 22002618

[B19] Ho KM Litton E Cost-effectiveness of using recombinant activated factor VII as an off-label rescue treatment for critical bleeding requiring massive transfusion Transfusion 2011 doi: 10.1111/j.1537-2995.2011.03505.x. [Epub ahead of print] 10.1111/j.1537-2995.2011.03505.x 22211634

[B20] Nascimento B Lin Y Callum J Recombinant factor VIIa is associated with an improved 24-hour survival without an improvement in inpatient survival in massively transfused civilian trauma patients Clinics (Sao Paulo) 2011 66 1 101 6 10.1590/S1807-59322011000100018 21437444 PMC3044583

[B21] Rizoli SB Nascimento B Jr Osman F Recombinant activated coagulation factor VII and bleeding trauma patients J Trauma 2006 61 6 1419 25 10.1097/01.ta.0000243045.56579.74 17159685

[B22] David Recombinant Activated Human Factor VII (NovoSeven) http://www.canadianmedicine4all.com/recombinant-activated-human-factor-vii-novoseven.html

[B23] Stein DM Dutton RP Hess JR Low-dose recombinant factor VIIa for trauma patients with coagulopathy Injury 2008 39 9 1054 61 10.1016/j.injury.2008.03.032 18656871

[B24] Karkouti K Beattie WS Arellano R Comprehensive Canadian Review of the Off-Label Use of Recombinant Activated Factor VII in Cardiac Surgery Circulation 2008 118 4 331 8 Epub 2008 Jul 7 10.1161/CIRCULATIONAHA.108.764308 18606914

[B25] James I John M Australia and New Zealand Haemostasis Registry 2010 Monsah University, Australia

[B26] Hess JR Brohi K Dutton RP The Coagulopathy of Trauma: A Review of Mechanisms J Trauma 2008 65 4 748 54 Review 10.1097/TA.0b013e3181877a9c 18849786

[B27] Knudson MM Cohen MJ Reidy R Trauma, Transfusions, and Use of Recombinant Factor VIIa: A Multicenter Case Registry Report of 380 patients from the Western Trauma Association J Am Coll Surg 2011 212 1 87 95 Epub 2010 Nov 5 10.1016/j.jamcollsurg.2010.08.020 21115374

[B28] CRASH-2 Trial Collaborators Effects of tranexamic acid on death, vascular occlusive events, and blood transfusion in trauma patients with significant haemorrhage (CRASH-2) a randomized, placebo-controlled trial Lancet 2010 376 9734 23 32 Epub 2010 Jun 14 20554319 10.1016/S0140-6736(10)60835-5

[B29] Guerriero C Cairns J Perel P Cost-effectiveness analysis of administering tranexamic acid to bleeding trauma patients using evidence from the CRASH-2 trial PLoS One 2011 6 5 e18987 10.1371/journal.pone.0018987 21559279 PMC3086904

[B30] Charoencholvanich K Siriwattanasakul P Tranexamic Acid Reduces Blood Loss and Blood Transfusion after TKA: A Prospective Randomized Controlled Trial Clin Orthop Relat Res 2011 Epub ahead of print 10.1007/s11999-011-1874-2 PMC3171556 21512813

[B31] Sepah YJ Umer M Ahmad T Use of Tranexamic acid is a cost effective method in preventing blood loss during and after total knee replacement J Orthop Surg Res 2011 6 1 22 10.1186/1749-799X-6-22 21600028 PMC3117744

